# Chemical structure metagenomics of microbial natural products: surveying nonribosomal peptides and beyond

**DOI:** 10.3762/bjoc.20.253

**Published:** 2024-11-20

**Authors:** Thomas Ma, John Chu

**Affiliations:** 1 Department of Chemistry, National Taiwan University, Taipei City 10617, Taiwan

**Keywords:** bioinformatics, chemical structure metagenomics, natural products, natural product discovery, nonribosomal peptides

## Abstract

Bioactivity-guided fractionation (BGF) has historically been a fruitful natural product discovery workflow. However, it is plagued by increasing rediscovery rates in recent years and new methods capable of exploring the natural product chemical space more broadly and more efficiently is in urgent need. Chemical structure metagenomics as one such method is the theme of this Perspective. It emphasizes a chemical-structure-centered viewpoint toward natural product research. Key to chemical structure metagenomics is the ability to predict the structure of a natural product based on its biosynthetic gene sequences, which facilitated the discovery of numerous new bioactive molecules and helped uncover oversampled/underexplored niches of decades of BGF based discovery. While microbial nonribosomal peptides have been the focus of chemical structure metagenomics efforts thus far, it is in principle applicable to other natural product families. The future outlook of this new approach will also be discussed.

## Introduction

Natural products present diverse structures to elicit a wide range of biological activities and are of paramount importance to both translational science and basic research. Despite not knowing the underlying active ingredient(s) and their mechanisms of action, humans have taken advantage of the therapeutic effects of herbal and microbial extracts for thousands of years [1]. We now know that natural products, genetically encoded secondary metabolites, are in most cases responsible for the desirable effects in these traditional medicine formulations [2]. Modern science has found that natural products are uniquely suited as the starting point for the development of new drugs [3–4]. They may serve as chemical probes and help confirm laboratory findings [5]; furthermore, entirely new physical and chemical principles are sometimes uncovered as a result of natural product research [6–9].

**Figure 1:**
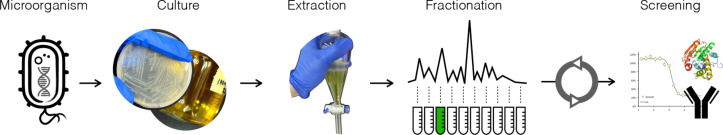
In BGF for microbial natural product discovery, the culture extract is fractionated using chromatographic methods. Fractionation is an iterative process guided by screening for the desired bioactivity until the isolation of a pure compound. This workflow was invented by Selman Waksman and has facilitated the identification of the vast majority of natural products known to date. However, it has fallen out of favor due to unacceptably high rediscovery rates, because BGF has nearly exhausted the rather limited chemical space it can access.

Two milestone achievements ushered in the modern era of natural product research. The first was the serendipitous discovery of penicillin in 1929 by Alexander Fleming, which went on to save millions of lives [10]. The second was the invention of the bioactivity-guided fractionation (BGF) workflow by Selman Waksman to search systematically for new bioactive small molecules (Figure 1) [11]. He believed that the fungus *Penicillium rubens* produces penicillin to suppress the growth of other microorganisms, especially those it competes with for nutrient and space, and that “chemical weapons” of this sort are widespread in nature. Waksman used the BGF workflow to find more than 20 new antibiotics throughout his career, wherein microbial culture extracts were fractionated and then tested for antimicrobial activity. The active fractions would be fractionated further, tested again, and this process would be performed iteratively until a pure compound is obtained. Notably, BGF is not limited to the identification of metabolites with antimicrobial activity or those of bacterial origin. It is applicable to the screening of natural products of almost any bioactivity of interest that originate from plants, fungi, and even animals (such as sponges and corals).

## Perspective

### BGF can access only a fraction of the natural product chemical space

For nearly a century, BGF has been the method of choice and identified the vast majority of natural products known to date. However, not long after the extensive application of BGF, scientists noticed an unwanted trend, i.e., it became more and more likely to isolate a natural product that has already been characterized, also known as “rediscovery” [4]. As rediscovery amounts to nothing but wasted time and money, BGF faced a diminished return-on-investment problem and gradually fell out of favor. This problem can be attributed to the fact that BGF nearly exhausted the rather limited natural product chemical space it can access [12–13]. Indeed, it has long been known that only a fraction of microorganisms is readily cultured in the laboratory [14–16]. More recently, large-scale sequencing studies and bioinformatic analyses estimated that BGF-based discovery covered only ≈1% of the biosynthetic diversity nature has to offer [17]. This is because there are two pre-requisites for a natural product to be amenable to the BGF workflow. Not only the microorganism of interest needs to be readily cultured, it must actively express its natural product biosynthetic gene cluster(s) (BGC) under laboratory culture conditions [18–19]. It turns out these pre-requisites are rarely met [16].

### New approaches to enable broader exploration

**Figure 2:**
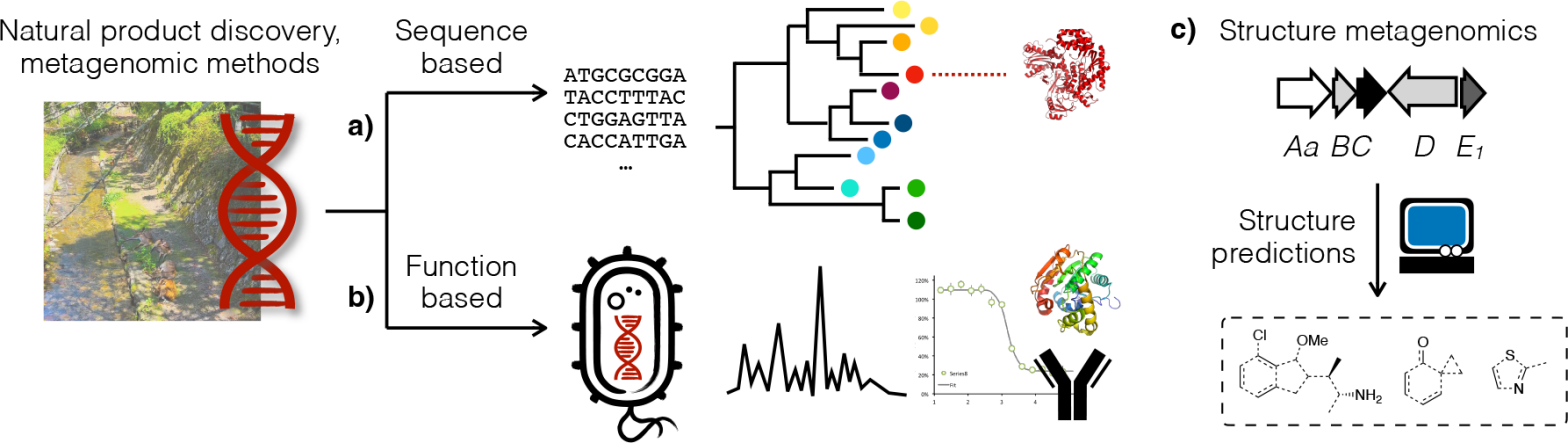
In light of BGF’s decreasing return-on-investment, scientists have developed new natural product discovery methods to tap into the microbial biosynthetic diversity of the “uncultured majority”. Sequence metagenomics and function metagenomics methods are reviewed elsewhere [26–28]. This Perspective focuses on chemical structure metagenomics, an emerging field founded on our ability to predict the chemical structure of natural products based on the sequences of their biosynthetic genes.

New approaches have been developed to circumvent the aforementioned constraints and enable the exploration of a broader natural product chemical space. Existing approaches fall into two broad categories – sequence metagenomics (Figure 2a) and function metagenomics (Figure 2b). The former analyzes nucleic acid sequences to prioritize BGCs that are worth exploring [20]. For example, it has been shown that tracking characteristic biosynthetic or self-resistance gene(s) can facilitate the discovery of new congeners of a natural product family [21–22]. Function metagenomics uses genetically tractable model organisms, such as *Escherichia coli* or *Streptomyces albus*, to express DNA extracted from the environment and then screen for the phenotype of interest [23–24]. This approach was used to identify BGCs that produce antibiotics, pigments, compounds that alter the morphology of the host, etc. [25]. Sequence and function metagenomic approaches have been reviewed elsewhere and will not be covered herein [26–28].

This Perspective focuses on chemical structure metagenomics (Figure 2c), an emerging field that integrates bioinformatics, chemical synthesis, molecular biology, etc., and emphasizes a chemical-structure-centered viewpoint toward natural product research. It should be noted that these metagenomic approaches are not mutually exclusive; rather, they complement each other and together provide a more complete picture. Chemical structure metagenomics approaches have already facilitated the discovery of numerous new bioactive molecules [29–36] and shed light on the scope of past discovery efforts by uncovering select oversampled/underexplored niches in the natural product chemical spaces [37]. While nonribosomal peptides (NRP) from microorganisms have been the focus of chemical structure metagenomics approaches thus far, it is in principle applicable to other natural product families as well, and the possibility of extending it beyond NRPs will be discussed at the end.

### Chemical structure is the universal language of nature

The chemical structure of a molecule is defined as the arrangement of its atoms and bonds, which describes not just the size and shape of a molecule, but also encompass its stereochemistry, charges, polarity, functional groups, as well as the way these elements are spatially oriented. The chemical structure of a molecule defines its properties and reactivity, and therefore dictates how it interacts with other molecules. Microorganisms need to communicate with each other and the environment, secreting signals of friendship, disdain, and many other sentiments in between. Natural products are the vocabulary for this molecular language, and knowing their chemical structures is key to understanding this form of information exchange [38].

It is worth pointing out that chemical structure, but not bioactivity, is the unique descriptor of a molecule. Molecules with the same chemical structures are (by definition) identical; they must act on the same cellular target and display the same bioactivity. It is not necessarily the case the other way around for bioactivity. However, bioactivity has long been used as a proxy to help find new natural products, whose chemical structure is typically determined after it has been isolated via the lengthy iterative purification process guided by bioactivity. Rediscovery is inevitable based on such a workflow, and this is essentially due to our inability to predict the chemical structure ahead of time. Does this have to be the case?

The instructions for natural product biosynthesis, along with all the information an organism needs, are encoded in its genome. Genetic information is transcribed and translated into proteins that carry out chemical reactions that sustain life, which include both primary metabolism and the biosynthesis of natural products. Direct protein sequencing by Edman degradation used to be standard practice in studying proteins but nowadays is rarely performed [39]. This is because the rules of transcription and translation are understood well enough for the sequence of a protein (its primary structure) to be predicted based on the corresponding nucleic acid sequence. Proteinaceous enzymes then go on to catalyze biosynthetic reactions that put together small molecule building blocks (BB) to generate natural products with extremely diverse chemical structures. Because the intricacy of this process is not fully understood, scientists still need to wait for enzymes to complete the entire course of biosynthesis, and then characterize chemical structure of the final natural product. While a generalized algorithm is not yet available, scientists in recent years have made some headways toward predicting the chemical structure of a natural product. Specifically, it is now possible to predict the order and identity of the BBs in NRPs based solely on the nucleic acid sequences of its BGC [40–49]. These algorithms not only obviated the need for culture and gene expression, dereplication can now be done in silico to avoid rediscovery. They are the cornerstone of chemical structure metagenomics; a few examples in this area of research are described below.

### NRP biosynthesis and structure prediction

**Figure 3:**
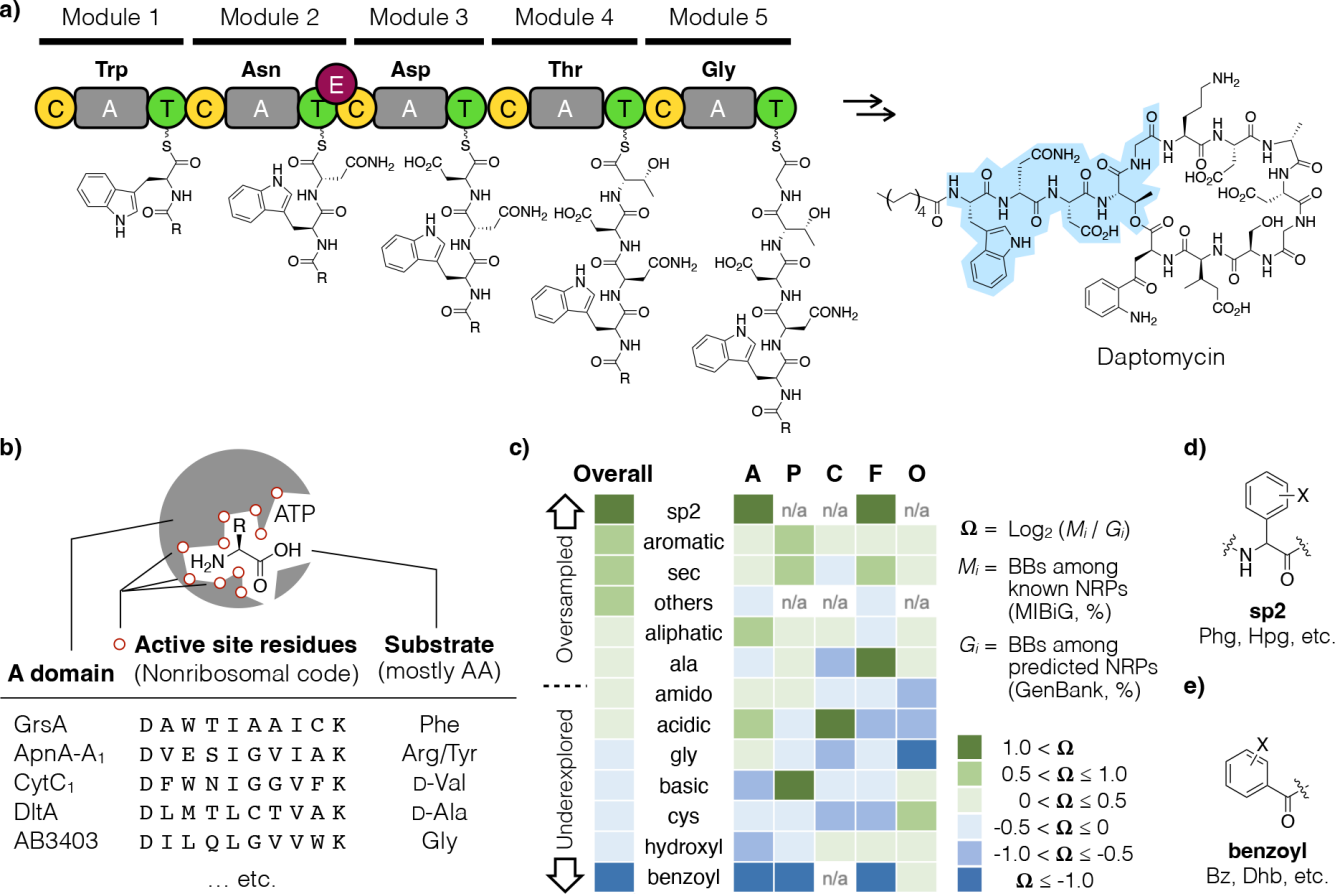
a) Incorporation of the first five amino acid BBs in daptomycin (highlighted in blue) is illustrated herein. In NRP biosynthesis, modules are arranged in an assembly line fashion; each module incorporates one BB into the growing peptide intermediate, which is then passed onto the next module. b) Within each module, the A domain (gray rectangles in a) is responsible for activating the substrate BB to be incorporated, and the substrate BB specificity is dictated by the nonribosomal code (the 10 residues that defines its binding pocket, small red circles). Algorithms for A domain substrate prediction were trained based on a dataset of known pairs of nonribosomal code/substrate BB. c) An analysis that compared the known vs predicted NRP BBs grouped based on their chemical structures found both oversampled (green)/underexplored (blue) niches in the NRP chemical space; the analysis was performed across all bacteria (overall) and for the top four most common phyla (Actinobacteria (A), Proteobacteria (P), Cyanobacteria (C), Firmicutes (F), and others (O)). d) and e) are the most oversampled and underexplored groups of NRP BBs, respectively.

NRPs are biosynthesized by either type I or II nonribosomal peptide synthetase (NRPS) [50–51]. Type I NRPS is a megaenzyme machinery that contains multiple modules arranged in an assembly line fashion, each of which is responsible for incorporating a single BB into the growing peptide intermediate (Figure 3a). One module typically contains one adenylation (A) domain that folds and operates semi-autonomously, which recognizes and activates a specific substrate BB. Notably, the BBs are in most cases amino acids, wherein a much broader variety are used in NRP biosynthesis than the 20 canonical amino acids used in protein biosynthesis [52]. The modules are usually arranged co-linearly to the BGC sequence, which makes bioinformatic analysis much more straightforward, and for this reason, NRPs had been the target of early efforts aimed at predicting the chemical structure of natural products based on biosynthetic gene sequences.

In 1999, Stachelhaus et al. reported that the substrate specificity of an A domain, i.e., the identity of the amino acid BB it recognizes and activates, can be predicted based on its gene sequence alone [40]. Numerous prediction algorithms of this kind have been reported since [40–49]. The A domains are highly conserved in terms of both structure and sequence [41,53–57]. The exception is the 10 residues that constitute the A domain active site, whose high variability creates binding pockets of varying shapes and sizes. These residues therefore dictate substrate BB specificity of an A domain and are referred to as the nonribosomal code (in analogy to the genetic code) [58]. Hundreds of known nonribosomal codes and their corresponding BBs can be extracted from natural products that have been characterized over the past several decades, generating a dataset to train NRP prediction algorithms (Figure 3b) [49,59–60]. A software suite called antibiotics and secondary metabolite analysis shell (antiSMASH) further automated all of the following steps: take (meta-)genomic sequences as the input, identify BGCs for NRPs (and other natural products as well), parse out modules and domains, and finally outputs the order and identity of BBs of a predicted NRP. AntiSMASH is freely available for the research community worldwide [61].

### Underexplored and oversampled NRP building blocks

With these prediction tools at hand, it became possible to survey the biosynthetic diversity of NRPs from a new perspective. Specifically, Jian et al. compiled data in GenBank into a custom microbial genome collection, termed GB1 [37]. Very little is known about most GB1 microorganisms aside from their genome sequences, much less their potential ability to produce natural products. On the other hand, MIBiG is the most comprehensive collection of known natural products whose BGCs have been sequenced and annotated [59]. As mentioned above, even though BGF found the vast majority of natural products we know thus far, they collectively account for a meager ≈1% of the chemical space. It begs the question of whether BGF has been sampling evenly or was there any bias. In contrast to BGF only being amenable to actively expressed BGCs in readily cultured microorganisms, there are very few pre-requisites to DNA sequencing, and therefore GB1 represents a (nearly) even sampling of the universe of microbial biosynthetic diversity.

Jian et al. applied A domain prediction algorithms to genome sequences in MIBiG and GB1. They estimated the relative abundance of predicted BBs among known NRPs (MIBiG) versus the predicted NRPs (GB1). The Ω parameter they presented was based on the log2 scale, wherein Ω = +1 and −1 means BGF-based natural product research has oversampled and underexplored by two-fold a particular BB (or a group of BBs with similar chemical structures), respectively (Figure 3c). Phenylglycine and its derivatives (the **sp2** group of BBs, Figure 3d) turned out to be the most oversampled group of BBs. This group of BBs included noncanonical amino acids characteristic of the glycopeptide antibiotic family [62–63]. Their aromatic rings undergo oxidative coupling to form biaryl moieties that restrict atropisomerism; the resulting rigid structure is key to their high affinity binding to peptidoglycan intermediates [64–65]. Glycopeptide antibiotics include vancomycin, teicoplanin, ramoplanin, etc., and were once an intense research focus in both academia and industry [66]. Such a historical backdrop may explain the apparent oversampling of the **sp2** BBs. In contrast, hydroxylated benzoic acids turned out to be the most underexplored group of BBs (**benzoyl**, Figure 3e). In NRPs, they serve as ligands for chelating iron in siderophores. Because ferric cations (Fe^3+^) mostly exist as insoluble solids in the Earth crust, most microorganisms produce and secrete siderophores to scavenge this scarce resource from their surroundings [67]. Whereas siderophores do not always contain hydroxylated benzoyl BBs, all known NRPs that do contain the benzoyl BB are siderophores. One plausible interpretation for this group of BBs being the most underexplored is that there are as yet unknown benzoyl BB containing NRPs that play other biological roles; we may have thus far completely missed them. Together, this chemical structure metagenomic analysis showed that BGF has not examined the natural product chemical space evenly, as there are niches that have been examined more frequently than random sampling, and there appear to be many stones left unturned as well.

### Converting predicted NRPs into real molecules

**Figure 4:**
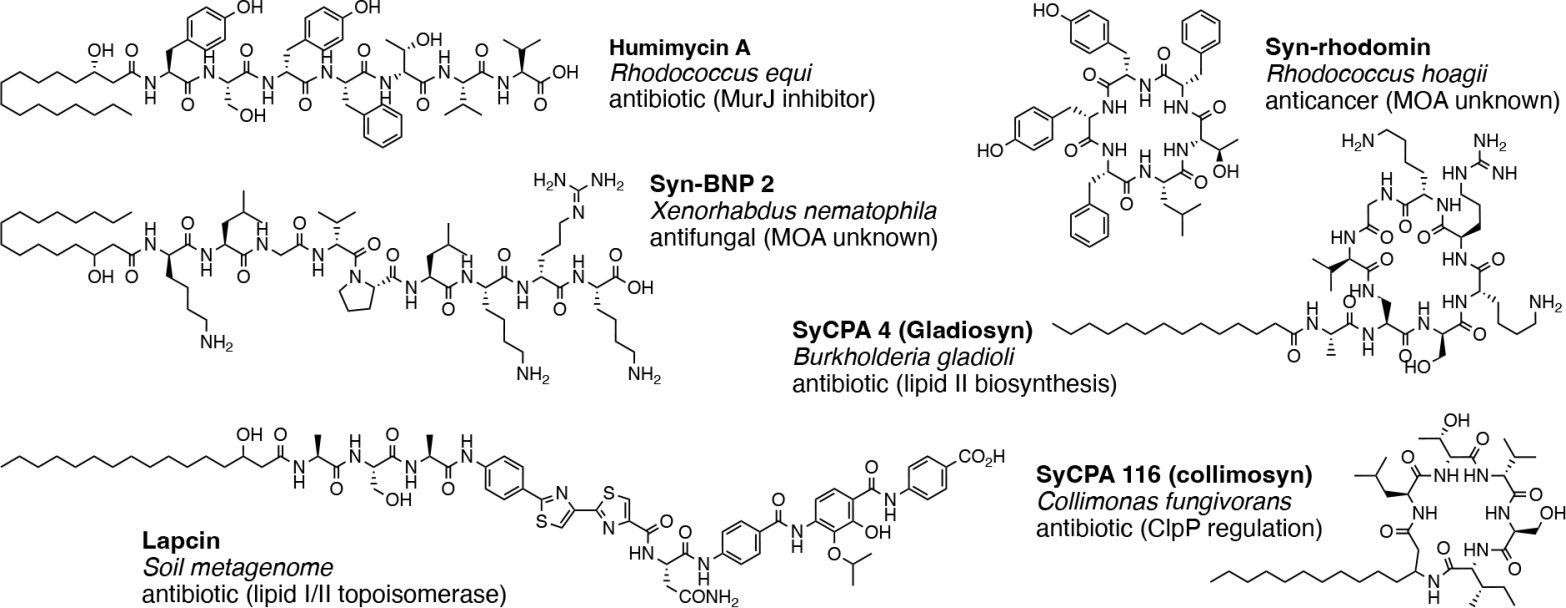
Syn-BNPs were synthesized in accordance to predicted NRP structures; shown herein are hits from various bioactivity screens. For each Syn-BNP, its name is in bold, the microorganism that harbors the corresponding BGC is italicized, followed by its bioactivity and the target/mechanism of action (if known).

Aside from an analysis of the BB usage pattern, real NRP-like molecules can be constructed in accordance to the predicted identity and order of the BBs. Brady and co-workers used solid-phase peptide synthesis to convert the predicted NRPs from virtual into reality; they called these molecules synthetic-bioinformatic natural products (Syn-BNPs) [29–36]. By excluding Syn-NRPs that resemble known NRPs, they were able to focus on chemical spaces that have not been explored yet. Because many NRPs are produced and isolated as a collection of structurally similar congeners, it was believed that this approach shall be viable as long as a Syn-BNP bears enough resemblance to the natural counterparts to recapitulate their biological functions. More than 500 Syn-BNP NRPs were designed and synthesized based on the analysis of bacterial genome and metagenome sequences. These molecules were screened for various bioactivities and led to the discovery of new antibiotics, antifungals, as well as anticancer compounds (Figure 4).

Importantly, the underlying mechanism of bacterial growth suppression has been identified for several Syn-BNP antibiotics, which includes both general mode of action (MOA) (e.g., membrane lysis and depolarization) [30,34] and specific MOA (e.g., dysregulation of ClpP protease [33], inhibition of topoisomerase I/II [36,68], blocking lipid II transport by flippase [29], sequestration of cell wall biosynthetic intermediate C55-(di)phosphate, etc.) [35]. It is unlikely that a Syn-BNP is able to target a specific protein or pathway, unless it in fact recapitulated key structural feature(s) of a NRP. These observations are a testament to the feasibility of this approach. This approach has also been applied to focus on NRPs with particular physical properties. Specifically, Qian and co-workers examined 7395 bacterial genomes and identified more than ten thousand potential cationic NRPs. They focused on a few promising candidates after bioinformatic-based dereplication and found two NRPs, brevicidine and laterocidine, that worked in an animal thigh infection model [69].

### Future directions 1: Improve A domain prediction algorithms

NRP prediction algorithms are central to chemical structure metagenomics. Several mechanistically novel Syn-BNP antibiotics were discovered, and a systematic BB usage pattern analysis provided insights into oversampled/underexplored niches in NRP chemical space (see above). These success stories suggest that the existing algorithms for A domain substrate prediction were reasonably accurate. However, only about 6 out of 10 A domains were amenable to the current prediction algorithms. Jian et al. reported that ≈40% of A domains failed to match a nonribosomal code in the algorithm training dataset and were deemed “unpredictable” [37]. It is also possible that the A domain in question aligned so poorly to prototypical A domains that prevented the proper identification of the nonribosomal code itself [70]. Regardless of the scenario, these bioinformatically intractable A domains are distinct from known ones and point to enormous biosynthetic novelty that still awaits our exploration.

Compiling a dataset for training A domain substrate prediction algorithms has never been the objective for natural product research in the past. The current dataset has been the byproduct of cumulative NRP discovery, and its rate of expansion has been disappointingly slow. This is because new NRPs do not necessarily contain new A domains or new nonribosomal codes, so that the effective size of the training dataset does not always benefit from the discovery of a new NRP. Conversely, every “unpredictable” A domain, if its substrate specificity were to be experimentally determined, is guaranteed to be a new datapoint. In fact, various in vitro substrate characterization assays that studied A domains as stand-alone enzymes have been reported [53,57,71–77]. Investigating strategically these bioinformatically intractable A domains is a much more efficient way to acquire new datapoints, and the performance of A domain prediction algorithms should improve simply after re-training on an expanded dataset. These algorithms may also benefit from bioinformatic studies of adenylating enzymes in general; the discovery of a novel β-lactone biosynthesis pathway in *Nocardia* species is a good case in point [78].

### Future directions 2: Understanding thioesterase function

**Figure 5:**
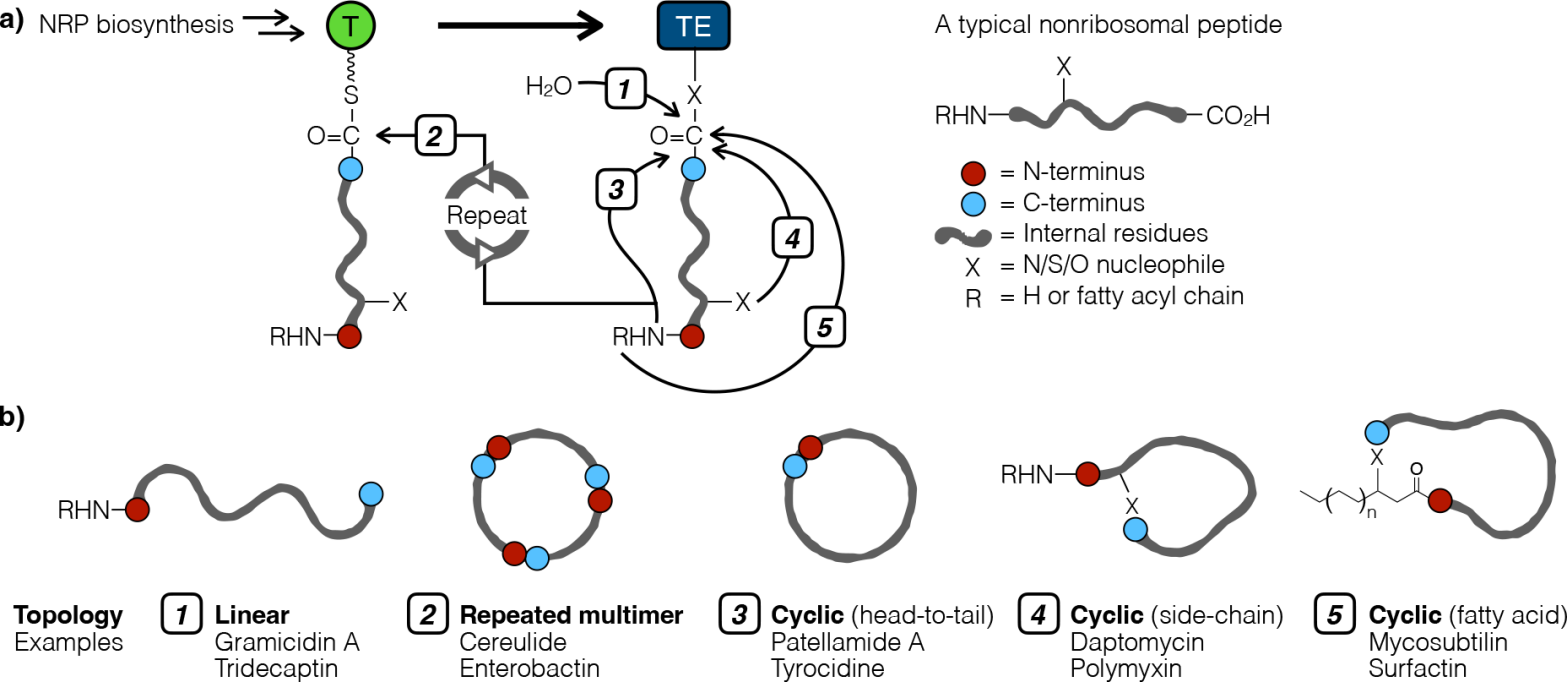
a) “Offloading” is the final step of NRP biosynthesis, wherein the mature NRP is released from the enzymatic assembly line via a thioesterase (TE)-catalyzed nucleophilic attack. Depending on the nature of the nucleophile, this reaction may generate distinct NRP topologies and the five most common types are shown above. b) Cartoon illustration of the five most common NRP topologies and two well-known examples for each category.

No algorithm is currently capable of predicting the topology of an NRP despite the fact that this feature is known to be important for bioactivity [79–80]. Typically, the C-terminus of the NRP intermediate is covalently linked via a thioester bond to the phosphopantetheine prosthetic arm of the peptide carrier protein (also known as the thiolation (T) domain) throughout biosynthesis (Figure 5a). The NRP intermediate is passed from one module to the next as BBs are incorporated one at a time into this growing peptide chain. The very last step in NRP biosynthesis – offloading the final product from the enzymatic assembly line – is usually catalyzed by the thioesterase (TE) domain and determines the topology of the final NRP product. The TE may release the NRP as a linear peptide or cyclic peptide, and the latter further manifests many possible topologies (Figure 5b). In a nutshell, offloading is a TE-catalyzed nucleophilic attack to release an NRP from the megaenzyme machinery. Water can act as the nucleophile during the offloading step, which amounts to hydrolysis and results in a linear peptide. On the other hand, a cyclic peptide forms when an intramolecular functional group acts as the nucleophile in this step. Regardless of the resulting topology, the NRP offloading step always entails the same chemical reaction, wherein nucleophilic attack is promoted by the catalytic triad of a TE via general base catalysis. This is likely why traditional mechanistic studies that focused on the enzyme active site failed to work out how TEs control NRP topology.

A priori, two factors should be important in the offloading step. First, the biosynthetic command is undoubtedly encoded somewhere within the TE protein sequence. The second factor, one that has been overlooked, is the chemical structure of the NRP. Both of these factors may need to be included in an analysis to fully understand the determinant(s) of NRP topology. There are many ways to classify NRPs when topology and chemical structural features are considered simultaneously. For example, a macrocycle may be a lactam or a lactone depending on whether the internal nucleophile is an amine or an alcohol, respectively. Based on the position of the nucleophile, a NRP can be cyclized head-to-tail, via an amino acid side-chain, a nucleophilic heteroatom on the N-terminal fatty acyl chain, or as a multimer of repeating sub-structures (Figure 5b). The ring size, ratio of ʟ- and ᴅ-amino acids, etc. may also be the basis of classification. TE sequence alignment guided by NRPs grouped based on one or more of the above topological features may yield key insights.

### Future directions 3: Chemical structure metagenomics of type I polyketides

The two largest natural product families are NRP and polyketides (PKs) [81]. Type I PKs are also constructed in a modular assembly line biosynthetic logic and may be amenable to chemical structure metagenomic approaches described herein [82]. In fact, the software suite antiSMASH can already predict the substrate specificity of individual polyketide synthase (PKS) modules, i.e., the type of alkylmalonate it incorporates into a growing PK intermediate [61]. Furthermore, Xiang et al. reported recently that the stereochemistry of each new chiral center resulting from each alkylmalonate BB incorporation can be predicted based on the corresponding ketoreductase domain sequence [83]. Last but not least, predicted polyketide structures can be synthesized in a streamlined and automated fashion by using a microfluidic device reported by Burke and co-workers [84–85]. As such, the necessary computational and synthetic tools are all in place to examine the PK chemical space and to support a Syn-BNP based PK discovery campaign.

### Future directions 4: Natural products with more complex biosynthetic logic

Type I NRPs and PKs, as well as many sub-families of ribosomally synthesized and post-translationally modified peptides (RiPPs), are readily amenable to chemical structure metagenomics studies, because translating their modular biosynthetic logic into a chemical synthetic scheme is rather straightforward [86]. Aside from constructing the core molecular scaffold of these natural products, further modifications may be installed either before or after the assembly of the amino acid or alkylmalonate BBs. While predicting most modifications de novo is not yet built into existing algorithms, it is often possible to work them out based on chemical structure context, background knowledge, and educated guesses. For example, cyclodehydration, a feature frequently seen in both NRPs and RiPPs [87], requires the presence of a nucleophile at the β-position of the amino acid and occurs exclusively on select amino acids. Specifically, cyclodehydration of a serine or threonine (followed by oxidation or reduction) generates an oxazole, oxazoline, or oxazolidine moiety in the NRP backbone, and the analogous thiazo moieties come from cysteines. The same requirement applies to the formation of dehydroalanine and dehydrobutyrine moieties. These chemical principles hold true even though the tailoring enzymes for these modifications in NRPs and RiPPs are nonhomologous [88–89].

Some types of modifications are biased towards (or against) certain amino acids; while these trends are statistically valid, whether there is an underlying chemical principle that governs the observed selectivity remains unclear [52]. For example, tailoring enzymes for β-hydroxylation most often act on aspartate and asparagine. In contrast, despite being one of the most common BBs in NRPs, no ornithine is β-hydroxylated to the best of our knowledge.

Last but not least, natural product research has forayed into the use of artificial intelligence (AI) tools. For example, Magarvey and co-workers used machine learning to help improve bioinformatic analysis. Their updated PRISM 4 pipeline outperformed antiSMASH5 and was able to predict the chemical structures of a wide variety of secondary metabolites [90]. Notably, the repertoire of PRISM 4 spans beyond NRPs and PKs and includes alkaloids, terpenoids, aminoglycosides, nucleosides, etc. Structure prediction of natural products other than NRPs and PKs has historically progressed slower. However, it was not due to a lack of data. Indeed, terpenoids and alkaloids are the two largest families of natural products in plants and also represent a sizable minority in microorganisms. The lagging progress is in fact due to our inability to interpret the corresponding genetically encoded biosynthetic instructions. While scientists will undoubtedly find more applications of AI, its strongest suit is arguably to help humans find hidden patterns in bulk data, such as the many bits and pieces of information that are available for terpenoids and alkaloids biosynthesis. Data standardization (albeit a tedious and labor-intensive task) may be the final roadblock between AI and generalizable chemical structure metagenomics [91].

## Conclusion and Outlook

Microorganisms produce specialized metabolites to communicate with each other and to interact with the environment. The chemical structure is the unique descriptor of these molecules and dictates the way these molecules behave and interact. However, the research and discovery of natural products have historically been guided by bioactivity and not chemical structure. We argue that, whether it is for the purpose of finding new lead compounds for drug development or gaining a deeper understanding in life science, the field of natural product research may benefit from placing chemical structure front and center. A few examples are described herein to illustrate the power of this viewpoint. It complements existing approaches to facilitate a broader and more efficient survey of the vast natural product chemical space that awaits our exploration.

## Data Availability

Data sharing is not applicable as no new data was generated or analyzed in this study.
